# The Role of Fibroblast Growth Factors in Tooth Development and Incisor Renewal

**DOI:** 10.1155/2018/7549160

**Published:** 2018-03-11

**Authors:** Wen Du, Wei Du, Haiyang Yu

**Affiliations:** ^1^State Key Laboratory of Oral Diseases, National Clinical Research Center for Oral Diseases, Department of Prosthodontics, West China Hospital of Stomatology, Sichuan University, Chengdu 610041, China; ^2^State Key Laboratory of Oral Diseases, National Clinical Research Center for Oral Diseases, Department of Endodontics, West China Hospital of Stomatology, Sichuan University, Chengdu 610041, China

## Abstract

The mineralized tissue of the tooth is composed of enamel, dentin, cementum, and alveolar bone; enamel is a calcified tissue with no living cells that originates from oral ectoderm, while the three other tissues derive from the cranial neural crest. The fibroblast growth factors (FGFs) are critical during the tooth development. Accumulating evidence has shown that the formation of dental tissues, that is, enamel, dentin, and supporting alveolar bone, as well as the development and homeostasis of the stem cells in the continuously growing mouse incisor is mediated by multiple FGF family members. This review discusses the role of FGF signaling in these mineralized tissues, trying to separate its different functions and highlighting the crosstalk between FGFs and other signaling pathways.

## 1. Introduction

Organogenesis is a complex physiological process. An intricate array of signaling molecules such as FGFs, bone morphogenetic proteins (BMPs), Wnt, and Hedgehog (Hh) families are known to regulate the formation, differentiation, and maintenance of the tooth and alveolar bone during the development and throughout adulthood [1–4].

FGF signaling occupies a significant position in inducing the proliferation and differentiation of multiple cell types during embryonic stages [5–10], as well as in regulating the development in different animals [11–14]. In addition, FGFs have been shown to regulate mouse tooth development [2, 15–17]. Nevertheless, a comprehensive description about the mechanism underlying FGFs that regulate different mineralized tissues of tooth during the embryonic stages, as well as incisor renewal in the adulthood, is still needed. Here, we summarize the roles of FGF signaling in mouse tooth development and the ways FGFs control the stem cells in incisor renewal, trying to separate its different functions and highlighting the crosstalk between FGFs and other signaling pathways.

## 2. Development of Tooth and Supporting Bone Structure

Most vertebrate groups have the ability to replace their teeth. Mammals have two sets of teeth: primary and adult teeth. In contrast, mice contain one set with two different types: molars located at the proximal area and incisor located at the distal area, which are separated by the toothless diastema region. Mouse incisors grow continuously throughout the lifetime in sharp contrast to the molars. It has been demonstrated that the presence of stem cells, which are located in the proximal end of the incisor, gives rise to the differentiated tooth cell types, thus promoting continuous growth of this tooth [18].

It has been widely held that tooth morphogenesis is characterized by the sequential interactions between the mesenchymal cells derived from the cranial neural crest, and the stomadial epithelium [19, 20]. This process consists of several phases, that is, bud, cap, and bell stages. In mice, the dental mesenchyme is attributed to neural crest cells which are derived from the midbrain and hindbrain regions around embryonic day 8.5 (E8.5) [21–24]. The determination of tooth-forming sites during E10.5 [25–27] and the thickening of the dental epithelium at E11.5 have been considered as the first signs of tooth development [28]. During the bud stage (E12.5–E13.5), in both incisor and molar, the thickened dental epithelium buds into the underlying mesenchyme, thus forming the epithelial tooth bud around the condensed mesenchymal cells. At the subsequent cap stage (E14.5–E15.5), the epithelial component undergoes specific folding. A central event, during the transitional process between bud and cap stages, is the formation of the enamel knot (EK), a structure composed of a group of nondividing cells. Moreover, several signaling molecules, such as Shh, FGF4, FGF9, BMP4, and BMP7, as well as Wnt10a/b, are restrictedly expressed in the enamel knot. Several studies have shown that the EK, as the signaling center, has an important role in tooth cusp patterning control [29, 30]. During the following bell stage, the ameloblasts and odontoblasts originate from the dental epithelium and mesenchyme, respectively [2]. At this stage, the secondary EKs (sEK) succeed the primary EKs (pEK) in the molar. In addition, the condensed mesenchymal cells around the developing epithelial tooth germ at the bud stage go on to differentiate into a supporting alveolar bone that forms the sockets for the teeth at the bell stage [31–33].

With reference to its origin, it has been reported that the alveolar bone is formed by intramembranous ossification [32, 33]. Intramembranous ossification starts with the mesenchymal cells which are derived from embryonic lineages correspondingly, which then migrate towards the locations of the future bones. Here, they form high cellular density condensations that outline the size and shape of the future bones. The mesenchymal cells subsequently differentiate into osteoblasts, thus forming bone directly within the condensations [3].

## 3. Stem Cells in Incisor Renewal and Osteogenesis

As it was previously mentioned, the adult mouse incisors can grow unceasingly throughout their lifetime, and this growth is counterbalanced by continuous abrasion. Essential to this phenomenon is the presence of active somatic stem cells which reside at the proximal end of the incisor. As a result, extensive studies have uncovered that the epithelial and mesenchymal stem cells of the incisor give rise to ameloblasts and odontoblasts, which are in turn responsible for producing new tissue which replaces worn enamel and dentin [1].

The epithelial stem cells reside in a niche called the cervical loop. From contemporary understanding of ameloblast development and maturation, these stem cells are located in the outer enamel epithelium (OEE) and the stellate reticulum (SR) of the labial cervical loop. These stem cells give rise to the transit-amplifying (TA) cells, which are divided for several generations and then differentiate into preameloblasts. In turn, these cells give rise to mature ameloblasts that are characterized by three component stages: presecretory, secretory, and maturation zones [34]. In contrast, compared to the epithelial counterparts, the stem cells which are derived from the mesenchyme and reside in the dental pulp are relatively poorly characterized [1].

In addition to incisor renewal, stem cells also show powerful osteogenic potential due to their ability to differentiate into osteoblasts. For instance, the condensation of mesenchymal stem cells (MSCs) from the neural crest or mesoderm has shown to stimulate the beginning of mammalian skeletal development [4]. The alveolar bone tissue regenerates during the process of bone repair and synostosis after implantation, exodontia, and orthodontic treatment, indicating the importance of stem cells in bone repair and regeneration. Numerous techniques have been used to stimulate stem cell-driven osteogenesis [35], including direct implantation of undifferentiated cells, or after *in vitro* differentiation, as well as stimulation of native stem cell differentiation through cytokine introduction. Adult bone marrow-derived mesenchymal stem cells are potentially useful for craniofacial mineralized tissue engineering [36]. It has been shown that compared with conventional guided bone regeneration, implanted tissue repair cells induce regeneration of alveolar bone and decrease the need for secondary bone grafting [37]. Adipose-derived stem cells (ADSCs), like bone marrow stem cells (BMSCs) that are derived from the mesenchyme and provide a supportive stroma for cell differentiation, may be extensively used in osteogenesis. Yet, larger quantities of ADSCs may be harvested with less pain as opposed to BMSCs [38]. In the clinical setting, further investigations of optimization for stem cell harvesting as well as scaffold-based delivery are required given the challenges in stem cell transplantation [36].

## 4. FGFs and the Receptors

The mouse FGF family comprises 22 members and could be divided into seven subfamilies: FGF1 (FGF1 and FGF2), FGF4 (FGF4–6), FGF7 (FGF3, FGF7, FGF10, and FGF22), FGF8 (FGF8, FGF17, and FGF18), FGF9 (FGF9, FGF16, and FGF20), FGF11 (FGF11–14), and FGF15 subfamilies (FGF15, FGF21, and FGF23) [39, 40]. FGF11 subfamilies (FGF11–14), also known as iFGFs, lack signal peptides and thus work as intracellular proteins. FGF15 subfamilies, consisting of FGF15, FGF21, and FGF23, are also known as hormone-like subfamilies (hFGFs) [41]. It is widely believed that iFGFs and hFGFs act in an FGFR-independent manner [42]. Other FGFs, which are also defined as canonical subfamilies, mediate their biological responses as extracellular proteins by binding to and activating cell surface tyrosine kinase FGF receptors (FGFRs) [39, 43]. FGFRs have been identified as four related transmembrane proteins comprising of a single transmembrane domain, an extracellular ligand-binding domain, and an intracellular tyrosine kinase domain [44].

*Fgfr1–3* undergo alternative mRNA splicing events and thereby generate alternative versions of the immunoglobulin-like domain III (IIIb or IIIc) [45]. This process increases the ligand-binding properties via regulation in a tissue-dependent manner [46–48]. The IIIb splice variant expression is predominantly detected in epithelial lineages and is responsible for transducing signals initiated by FGFs detected in the mesenchyme. Furthermore, the IIIc splice variant is restrictedly expressed in mesenchymal lineages and it transduces signaling from epithelial FGFs [49–53]. By contrast, *Fgfr4* is not alternatively spliced [54].

Triggered by the dimerization of receptors, the transphosphorylation and activation of FGFRs initiate signaling via multiple downstream intracellular pathways [55]. By binding to various arrays of adaptor proteins such as SHP2 and growth factor receptor-bound protein 2 (GRB2) [56–59], the activated receptor's cytosolic domain in turn mediates Ras signals to activate the downstream signaling cascades, such as PI3K/AKT and MAPK pathways [60].

While FGF signaling, encompassing FGF and FGFRs, occupies a critical position in regulating diverse cellular functions, it could be regulated by various upstream regulators. The most well-investigated regulator group are the Sprouty genes, which encode antagonists of FGF signaling by binding with GRB2 thus preventing Ras activation [61]. Other signaling pathways, for example, the Wnt pathway, have been recently identified as a positive regulator of FGF signaling [62].

## 5. Expression Patterns of FGFs during Tooth Development

**Figure 1:**
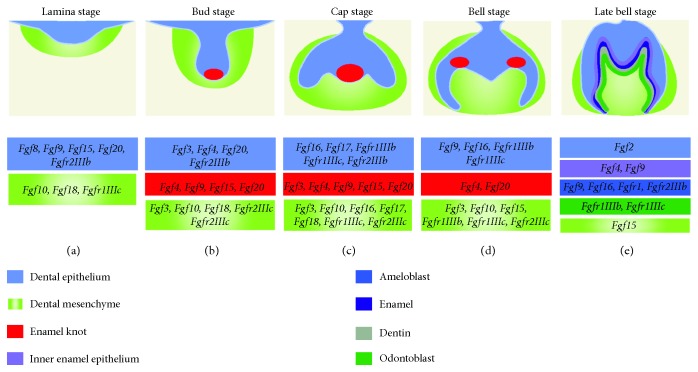
Schematic depiction of the expression of FGFs and the receptors in molar development. The lamina (a), bud (b), cap (c), bell (d), and late bell (e) stages of the mouse molar are shown in the frontal view.

FGFs are expressed in the dental epithelium throughout tooth development (Figure 1). During the initiation stage of odontogenesis, the expressions of *Fgf8*, *Fgf9*, *Fgf10*, *Fgf17*, and *Fgfr2IIIb* are detected in the prospective tooth region around E10.5 to E11.5 [63–66]. In the same region, following the formation of the dental lamina, *Fgf8*, *Fgf9*, *Fgf15*, and *Fgf20* are expressed, while the expression of *Fgf10* in the epithelium is decreased [63]. As the epithelial bud is formed unceasingly in the dental lamina, the *Fgf9* and *Fgf20* expressions persist while *Fgf3* and *Fgf4* are initiated [65, 66]. *Fgf3*, *Fgf4*, *Fgf9*, *Fgf15*, and *Fgf20* are expressed in the pEK after its formation, while the expressions of *Fgfr1IIIb*, *Fgfr1IIIc*, and *Fgfr2IIIb* are found in the dental epithelium. *Fgf16* and *Fgf17* are expressed in the cervical loop epithelium [65]. In the sEK at the bell stage, the *Fgf4* and *Fgf20* expressions are restricted in the forming cusps. The expressions of *Fgf9*, *Fgf16*, *Fgfr1IIIb*, and *Fgfr1IIIc* are detected in the differentiating ameloblasts. At the same time, the expressions of *Fgf1*, *Fgf9*, *Fgf16*, and *Fgf17* can be found in the cervical loop epithelium of the incisor [65, 66].

During tooth development, the expressions of FGFs are also detected in the mesenchyme (Figure 1). *Fgfr1IIIc* and *Fgf10* expressions are detected in the prospective tooth region during the early stage [63, 66]. During the thickening of the prospective tooth region epithelium which then forms the dental lamina, the expressions of *Fgf10* and *Fgf18* are found in the mesenchyme [63, 65]. After the formation of the epithelial bud, the expressions of *Fgf10* and *Fgf18*, as well as that of *Fgf3*, are found; besides, *Fgfr2IIIc* expression appears [65]. After pEK formation, *Fgf3*, *Fgf10*, and *Fgf18* are found in the mesenchyme [65]. The expressions of *Fgf16* and *Fgf17* are detected in the cervical loop mesenchyme while *Fgfr1IIIc* and *Fgfr2IIIc* are expressed in the mesenchyme of the buccal side [63, 65, 66]. At the late bell stage, *Fgf3* is expressed in the dental papilla, while *Fgf10* is expressed in the differentiating odontoblasts. In addition, *Fgf15* is restricted to the mesenchyme while the expressions of *Fgfr1IIIb* and *Fgfr1IIIc* are located in odontoblasts [63, 65]. Moreover, *Fgf3*, *Fgf7*, *Fgf10*, *Fgf16*, *Fgf18*, and *Fgf21* are also detected in the incisor [65].

The mesenchymal-derived alveolar bone is histologically detectable after E13.0, and its early formation occurs by E14.0. After E15.0, the development of the alveolar bone is well progressed. Comparative PCR array analysis has shown an increased statistical significance (14-fold) in the *Fgf3* expression levels between E13.0 and E15.0 [67]. In addition, *Fgf7* transcripts have been detected in the developing bone surrounding the tooth germ [63].

During tooth development, Sprouty (Spry) genes, as FGF antagonists, are also expressed in different tissues [68]. During the cap stage, the expression of *Spry1* appears in diastema buds and is highly expressed in the tooth germs of the first molar (M1), whereas *Spry2* is strongly expressed in the epithelium of both M1 tooth germ and diastema. *Spry4* is uniquely expressed in the mesenchyme in tooth germs of M1 and in the diastema. Nevertheless, *Spry3* is not detected within the tooth germ.

## 6. The Role of FGFs during Tooth Development

### 6.1. The Role of FGFs during the Formation of Enamel

Tooth formation begins with the first signals from the future tooth epithelium at E9.5 [69]. In the area where a prospective tooth forms, the oral ectoderm thickens; the epithelial *Fgf8*, *Fgf9*, and *Fgf17* expressions suggest that these FGFs may take part in the initiation of tooth development [65, 66]. An early study has shown that FGF8 can induce the expression of *Pax9* in mice, which reveals the prospective odontogenesis locations, and is essential beyond the bud stage of tooth development [25]. In the first branchial arch (BA1) with ectoderm Nestin-Cre, conditional *Fgf8* knockout leads to a decrease in *Pax9* expression in the expected molar region, and the formation of molar is stopped. The deletion of *Fgf8* does not affect *Pax9* expression within the presumptive incisor region, and thus the incisor is formed in a normal manner. The recent study has indicated that *Fgf8*-expressing cells labeled during the initiation stage of molars can furnish the epithelial cells and collectively migrate towards the dental lamina site which is important for prospective molar positioning [70]. In addition, the conditional deletion of *Fgf8* by E11.5 leads to an arrest in the formation of the dental lamina, and it also affects further development of the dental primordium and leads to a shorter invaginated structure [70]. At this early stage, *Fgf10*, a member from another FGF subfamily, is expressed in the epithelium [63]. Teeth develop in *Fgf10*-deficient mice, although a defect of the stem cell compartment in the incisor cervical loop has been observed [71], and deletion of *Fgf9* which is also expressed at the early stage does not affect tooth formation either [72, 73]. *Fgf17* expressed at the early stage is another member from the FGF8 subfamily. The expression of *Fgf17* occurs in the prospective molar rather than the incisor epithelium, indicating that FGF17 is involved in presumptive molar site positioning, like FGF8 [65]. It is believed that FGF8 is essential in determining the tooth type [25, 74], while FGF17 may also take part in this process. At E10, *Bmp2* and *Bmp4* offset the induction of *Fgf8* at the transcription level of *Pax9*, before dental ectoderm thickening. Furthermore, it has been shown that the initiation of odontogenesis only occurs in regions with the presence of the inducer FGF and the absence of its antagonists (BMPs), while the mesenchyme can react to the inducer.

The epithelium becomes thickened at the future tooth-forming site and subsequently forms the multilayered epithelium which then contributes to the dental lamina formation. The *Fgf10* expression is negatively regulated at this stage [63]. In the meantime, *Fgf8* and *Fgf9* are maintained in the epithelium. In the dental lamina, the initiation of *Fgf15* expression is detected on the lingual side whereas the expression of *Fgf20* is detected at the tip, implying that these FGFs participate in epithelial thickening [65]. Interestingly, it appears that the knockout of *Fgf9*, *Fgf10*, or *Fgf20* does not affect epithelial thickening or formation of lamina [73, 75]. This may result from the compensation between these FGFs, and the combination of conditional deletion at this stage is necessary to investigate the roles of these FGFs on lamina formation. In addition, *Fgf2rIIIb* is detected in the odontogenic epithelium at the early stage.

Subsequently, invagination of the dental lamina occurs in the underlying mesenchyme, while the cells in the mesenchyme condense around the dental epithelium, thus contributing to the formation of tooth bud and cap. FGF expression patterns suggest that the binding of FGF3 and FGF10 to FGFR2IIIb activate FGF signaling from the epithelium at the stages of invagination and tooth bud [64, 65]. In *Fgfr2*-deficient mice, the formation of tooth is inhibited after thickening of the epithelium. Although *Fgf3* and *Fgf10* in the mesenchyme can still be observed in *Fgfr2* mutants, the *Fgf3* expression in the epithelium is decreased [76].

Given that FGF3 and FGF10 bind to FGFR2IIIb, it is important for these FGFs to be involved in the transitional process to the tooth bud [77, 78]. Surprisingly, a single deletion of *Fgf3* or *Fgf10* in mice does not affect early tooth development, which proceeds normally to the cap stage. The deletion of both *Fgf3* and *Fgf10* has revealed that the development of molar is inhibited prior to the bud stage, suggesting possible compensations between *Fgf3* and *Fgf10* during invagination of the dental epithelium [79, 80]. At this stage, *Fgf9* is highly expressed in the tip of the bud. The deletion of *Fgf9* does not affect tooth bud invagination in mice; nevertheless, it affects progenitor cell differentiation in the incisor [72, 73]. The defective invagination of the dental epithelium in *Runx2*-deficient mice is recuperated by exogenous FGF9 protein [72, 81], which suggests that during tooth invagination FGF9 functions downstream of RUNX2 as an important factor. These results imply potential compensations between FGF9 and other FGFs in the epithelium. In addition, FGF9 upregulates *Msx1*, a homeobox-containing transcription factor essential for invagination of the tooth bud [66, 82].

During bud invagination, FGF signaling also regulates PITX2, an important transcription factor, whose expression in the oral epithelium is initially controlled by FGF8 and BMP4. FGF8 upregulates the expression of *Pitx2* whereas BMP4 represses it [83]. *Fgf8* expression in the oral epithelium decreases with the absence of *Pitx2* [84, 85]. In addition, the expression of *Fgf20* is restricted to the tip of the tooth bud. Early tooth development is not arrested in mice with deletion of *Fgf20* or *Fgf9* [73]. Considering these redundant roles, it would be useful to analyze double or triple FGF deletion to gain a better understanding of gene function at this stage. Recent study has shown that in the explant slice culture system, after treatment with a pan-FGF receptor inhibitor SU5402 at E11.5, a significantly shallower tooth bud has been detected. Interestingly, SU5402 treatment at E12.5 only results in narrower tooth bud formation, indicating that FGF signaling takes part in epithelium stratification but not placode invagination [86, 87]. This finding has been further complemented by gain-of-function experiments with FGF10-soaked beads towards the single-layered tongue epithelium [86, 87].

At the bell stage, FGF signaling is important in the differentiation of ameloblasts. The expressions of *Fgf4* and *Fgf9* are detected in the inner enamel epithelium (IEE) [66], while the expression of *Fgf2* is found in the SR, the expressions of *Fgfr1* and *Fgfr2IIIb* in the ameloblasts. With inactivation of *Fgfr1*, dysfunctional ameloblasts produce disorganized enamel [88]. In cultured embryonic molars, *Fgf2* overexpression leads to a decrease in amelogenin expression, whereas expression of amelogenin and formation of enamel increase with inhibition of FGF2 [89]. In tooth cultures, exogenous FGF2 and FGF4 promote the expression level of *Tbx1*, which can be expressed in the epithelium and encode a transcription factor. However, the expression of *Tbx1* decreases in *Fgfr2^−/−^* mice [90]. Besides, from in vitro cultured *Tbx1*-deficient mice, there is lack of ameloblasts while enamel is not formed in incisors, thus *Tbx1* is necessary for the differentiation of ameloblasts [91]. As downstream targets of FGFs, members of the Ras superfamily are also involved in amelogenesis. With conditional *Rac1* deactivation, a decreased level of amelogenin is expressed in ameloblasts, which also loosely attach to the secreted enamel matrix, and thus cause hypomineralization in enamel [92].

Decreasing Sprouty expression level can increase FGF signaling, which results in the formation of ectopic enamel and supernumerary teeth formation [68]. Ameloblast differentiation occurs and subsequently forms ectopic enamel on the lingual side of the incisor in *Spry2^+/−^;Spry4^−/−^* mice [93, 94]. Furthermore, HRas are downstream of FGFs and hypomineralization, and disorganization in enamel could be caused by increased HRas signaling in mice which could be rescued by inhibition of the MAPK pathway [95].

### 6.2. The Role of FGFs during the Formation of Dentin and Supporting Bone Structure

During the initiation stage, apoptosis occurs in mesenchymal cells in the BA1 proximal region with the absence of FGF8, which has an important role in survival of mesenchymal cells [96]. *Fgf10* is also expressed in the mesenchyme at this early stage [63]. As it was mentioned previously, the deletion of *Fgf10* in mice does not affect the formation of teeth [71], as well as FGF9 which is expressed in the epithelium at the same stage [72, 73]. Given these data, neither FGF9 nor FGF10 takes part in tooth site positioning. Another possibility is the redundant roles of these FGFs when the tooth initiates.

FGF18 is another member of the FGF8 subfamily. At the lamina stage, *Fgf18* expression is observed in the mesenchyme within the buccal side, unlike other FGFs from the FGF8 subfamily that are expressed in the epithelium. In tooth development, the function of FGF18 is still unknown, and further studies are necessary to determine its role in odontogenesis. Moreover, *Fgf1rIIIc* is found to be expressed in the mesenchyme at these early stages [66]. FGFs such as FGF2, FGF4, and FGF9 onto mandibular explants at this stage induce the expression of CCN2—one of the CCN proteins which are cell-associated and extracellular molecules relevant to several developmental processes—and can in turn promote dental mesenchymal proliferation [97].

Subsequently, dental lamina invagination takes place and mesenchymal cells condense to form a tooth bud and cap. In the mesenchyme during these stages, FGF4, FGF8, and FGF20 bind to FGFR1IIIc while FGF4, FGF8, FGF9, FGF16, FGF18, and FGF20 bind to FGFR2IIIc [64, 65]. Nevertheless, condensation of dental mesenchymal cells is not detected in *Fgfr2^−/−^* mice.

At the bud stage, the expression of FGF4 initiates in the epithelium. But in *Lef1*-null mice, the expression of *Fgf4* is reduced in tooth germs at E13, which in turn causes an arrest in mesenchymal condensation [98]. With exogenous FGF4, *Fgf3* expression is rapidly induced in mesenchyme and the defect in *Lef1^−/−^* tooth germs is fully rescued [99]. These data suggest that *Fgf4* may function as a transcriptional target gene of WNT signaling. At this stage, FGF18 is expressed in the mesenchyme, except for the region underneath the epithelium of the tooth bud. In order to understand the role of this FGF in odontogenesis, further studies are necessary [65].

During the cap stage and early bell stage, the expressions of *Fgf3*, *Fgf10*, and *Fgfr2* are detectable in the mesenchyme. Recent studies have demonstrated that *Twist1*, which is expressed in the mesenchyme, could bind to *Fgf10* and *Fgfr2* promoters and in turn regulate the *Fgf10* and *Fgfr2* expressions. In *Twist2*^*Cre*/+^;*Twist1^fl/fl^* mice, the expressions of *Fgf3*, *Fgf10*, and *Fgfr2* were significantly reduced at E14.5 and E15.5, indicating that FGF signaling could be affected by *Twist1* [100–102].

At the bell stage, the differentiation turns the cells from the dental papilla into odontoblasts, by which a dentin matrix is secreted. This matrix promotes differentiation which turns the epithelium into ameloblasts, which produce an enamel matrix [103]. The differentiation of odontoblasts is induced by FGFs from the EK [104, 105]. In addition, the expressions of *Fgf3* and *Fgf10* are found in the mesenchyme, and their expression is negatively regulated when dental papilla cells undergo differentiation to become odontoblasts [63, 106].

As mentioned earlier, the supporting alveolar bone is derived from condensed mesenchymal cells around the developing epithelial tooth germ, and it subsequently forms sockets for the teeth at the bell stage. During the formation of a molar root, FGF2 that is expressed in differentiating osteoblasts of the adjacent developing alveolar bone can stimulate the proliferation of chondrocytes, osteoblasts, and periosteal cells and stimulate the production of type I collagen [107]. FGF7, detected in the developing bone surrounding the molar tooth germ and the mesenchyme adjacent to the incisor cervical loop, is involved in the formation of alveolar bone [63]. Furthermore, the addition of FGF4 or FGF8 beads into mouse dental mesenchymal cells can promote their osteogenic differentiation and the expression of CBFA1, which belongs to the CBFA family and functions as an important regulator for differentiating osteoblasts in vertebrata [81]. Given the strong expression of CBFA1 in osteoblasts in tooth alveolar bone at the late bell stage, signaling of FGF4 and FGF8 from the epithelium may also have an important role during alveolar bone formation. It has also been reported that increased *β*-catenin signaling is related to the fate of dental mesenchymal cells, while FGF3 can sustain the odontogenic fate of incisor mesenchymal cells by downregulating intracellular *β*-catenin signaling [108]. Therefore, the lack of FGF3 could induce the potency of mesenchymal cells to differentiate into osteoblasts which are responsible for the formation of the supporting bone structure. Since the role of FGFs in supporting alveolar bone remains largely unexplored, further investigations are still needed.

### 6.3. The Role of FGFs in Tooth Size, Shape, Number, and Arrangement

The signaling center pEK, which regulates the size and shape of the tooth, consists of nonproliferative cells [109]. Different signaling molecules and their antagonists, including FGFs, Shh, Sprouty genes, BMPs, several WNTs, and follistatin, are expressed in pEK [110]. pEK cells cannot respond to FGFs since there are no FGF receptors expressed in these cells [66]. The nonproliferative cells in the pEK and the surrounding extensive proliferation cells may explain the epithelial folding and the transition process between the tooth bud and cap stages [15, 109]. Afterwards, the pEK induces the sEK in multicuspid teeth. The spatial arrangement of sEK has also been shown to contain a network of activators and inhibitors [111, 112]. The location and shape of the cusps are determined by the proliferation and differentiation of the epithelial cells which are regulated by the sEK; thus, the shape of the tooth crown is determined.

In molars, pEK size can affect the shape of the invaginated epithelium. Tooth size and cusp number decrease if the size of the pEK is too small, since a small size can affect the dental epithelium folding as well as the cervical loop and sEK formation. Ectodysplasin (Eda) and Traf6 are two members of the TNF-*α* family involved in tooth development regulation. A small size of the pEK will be present in mice without either of those proteins, and it will then result in reduced tooth size and cusp number [113, 114]. The arrangement of sEK will be changed in case signaling from the pEK is compromised by changing its size or shape; thus, defects of cusp will occur. Furthermore, molar shape and cusp patterns will be altered under modulation in the levels of gene expression in BMP, SHH, and WNT signaling [62, 115–119].

In the mesenchyme, the expression of *Fgf3* is maintained by FGF4 and FGF9, which are detected to be highly expressed in the pEK and sEK [63, 66]. FGF4 from the EK promotes the proliferation and has a role in the development of tooth cusps [30, 109]. Besides, FGF4 can also prevent cell apoptosis in the dental epithelium and mesenchyme [120, 121]. Nevertheless, inactivation of neither *Fgf4* nor *Fgf9* can affect tooth shape or number [72, 73]. Moreover, epiprofin, a transcription factor from the Sp family, can promote dental epithelial FGF9 which could elicit proliferation of dental mesenchymal cells through FGFR1c; this is essential for the tooth morphogenesis with the correct shapes and proper sizes [122].

FGF20 is another member of the FGF9 family, and its expression is found in the anterior bud of the lamina and the EK, along with the expressions of *Fgf3*, *Fgf4*, *Fgf9*, and *Fgf15* [65, 66, 123]. During tooth development, FGF20 functions as a downstream target of EDA: in *Eda* mutant mice, the *Fgf20* expression was reduced in molars, while it was increased in *Eda*-overexpressing (*K14-Eda*) mice [73]. In addition, *Fgf20* knockout mice exhibited molar teeth with reduced size and a mild change in the anterior cusp, while the overall pattern of the cusp was normal in *Fgf20* mutants. Therefore, FGF20 has shown to have a crucial role in fine tuning of the pattern of the anterior cusp and functions as a regulator of tooth size. Double knockout of *Fgf9* and *Fgf20* has shown strong additive effects by strikingly shortening the length of EK in comparison with the length of either single deletion mutant, which implies the redundancy between these two FGF ligands [73].

In the mesenchyme, FGFs have been shown to be involved in tooth shaping. Like *Fgf20*-deficient mice, *Fgf3^−/−^;Fgf10^+/−^* mice exhibit small molars [73, 80], and the *Eda^−/−^* molar phenotype can be partially offset by FGF10 *in vitro* [113]. Consequently, decrease in FGF signaling in either epithelium or mesenchyme can lead to similar effects during tooth formation.

Tooth number and arrangement are also found to be tightly regulated by FGF signaling within the dentition. Supernumerary teeth, which are mainly positioned at the prospective site of the premolar, have been found in several mutant mice. *K14-Eda* has been discovered as the first transgenic mouse line with ectopic teeth [124]. The following studies have reported that in this genetic background, the formation frequency of an extra tooth increased with lack of *Fgf20*, while single deletion of *Fgf20* could hardly promote the formation of an extra molar [73]. Supernumerary incisors and teeth anterior to the first molar have also been discovered in mice with deletion of Sprouty genes [68, 125]. To sum up, these findings indicate that FGFs function as stimulators, while Sprouty genes function as endogenous antagonists of FGF signaling in the development of the tooth.

## 7. The Role of FGFs in Incisor Stem Cell Renewal

It is well known that continuous growth of rodent incisor is counterbalanced by wear, which is promoted by the lack of enamel on the lingual side of the tooth surface. The absence of lingual ameloblasts results in the lack of enamel on that side [126]. Asymmetric wear maintains the length of incisor and leads to a sharp tip. The cervical loop includes various cell types: IEE cells, OEE cells, SR cells, TA cells, and stratum intermedium (SI) cells. In addition, an extra group of cells has been found between the SR and OEE [127]; however, their exact function still remains unknown.

**Figure 2:**
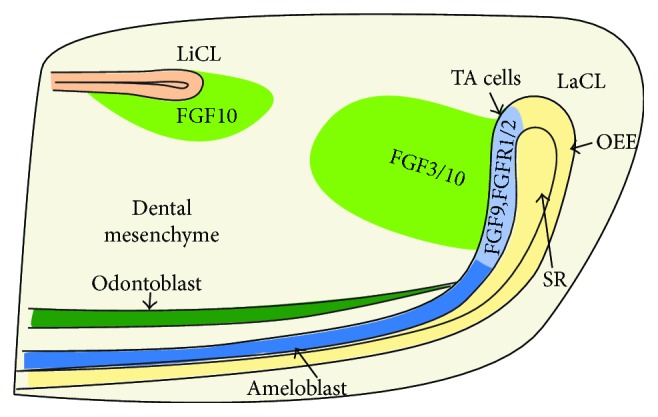
Expression patterns of FGF signaling molecules involved in the regulation of incisor cervical loop maintenance. *Fgf3* is expressed in the mesenchyme adjacent to LaCL, while *Fgf10* is expressed in the mesenchyme adjacent to both LaCL and LiCL. *Fgf9*, *Fgfr1*, and *Fgfr2* are restricted in transit-amplifying cells. LaCL: labial cervical loop; LiCL, lingual cervical loop; TA cells: transit-amplifying cells; OEE: outer enamel epithelium; SR: stellate reticulum.

FGF signaling is known to have an important role in the regulation of incisor cervical loop maintenance (Figure 2). During incisor development, an overlapping expression of *Fgf3* and *Fgf10* is initially detected in the dental papilla and is maintained through E14 in the incisor bud [79]. The expression of *Fgf10* remains stable in the mesenchyme adjacent to both labial and lingual IEE of the developing cervical loops from E16 to adulthood, while *Fgfr1b* and *Fgfr2b* are expressed in the forming cervical loops. *Fgf3* is the only protein expressed in the mesenchyme neighboring to the labial IEE [18, 63, 79, 80]. These FGFs expressed in mesenchyme are essential for the survival and proliferation of epithelial stem cells in the forming cervical loops; nevertheless, they are not essential for early ameloblast differentiation [79, 80]. This is consistent with the *Fgf10*^*−/*−^ embryos, whose cervical loop initially forms and then regresses due to increased apoptosis and decreased growth [79]. However, teeth in *Fgf3*-deficient mice are generally normal, which may result from the redundancy of *Fgf10*. Interestingly, *Fgf3^−/−^;Fgf10^+/−^* mutants develop a severely hypoplastic LaCL and either thin or missing enamel layer, suggesting that FGF signaling levels have an important role in the maintenance of the epithelial stem cell pool in the incisor [80]. Coincident with this result, mice without FGFR2IIIb have no distinct incisors at birth [77]. In addition, *Fgf9* is expressed in the epithelium of incisor [65, 66] and may function as a key factor in activating FGF expression in the mesenchyme [80, 128]. Consistent with this view, *Fgf3* and *Fgf10* in the dental mesenchyme are reduced with the genetic ablation of the core binding factor *β*, which in turn binds to *Runx* transcription factors and is essential for *Fgf9* expression in the epithelium [72]. FGF9 and FGF10 signaling both function through FGFR2b. The defect in ameloblasts and enamel, the suppression in *Shh* expression, and the decrease in cellular proliferation all occur with the conditional knockout of *Fgfr2b* or decrease in signaling via *Fgfr2b* [129, 130]. It coincides with the idea that in the cervical loop, the proliferation and differentiation of the progenitors are regulated by FGF9.

It has also been suggested that the spatial and quantitative balance of FGF signaling is important in maintaining the asymmetry of the incisor, where ameloblasts and enamel are located in the labial side. The intracellular antagonists encoded by *Sprouty* (*Spry1*, *2*, and *4*) are important regulators of FGF. As mentioned earlier, the expressions of *Sprouty* genes are detected in both labial and lingual epithelia and the adjacent mesenchyme [93]. In *Spry4^−/−^;Spry2^+/−^* mutants, both labial and lingual epithelial and mesenchymal cells reveal a large increase in sensitivity to FGF signaling. As a result, ectopic mesenchymal expressions of *Fgf3* and *Fgf10* as well as lingual ameloblast formation were observed [93]. The Sprouty genes may partially function by indirect regulation of BCL11B and TBX1, transcription factors which are, respectively, down- and upregulated in LiCL in *Spry4^−/−^; Spry2^+/−^* mutants at E16.5 [91, 106]. At E16.5, deletion of *Bcl11b* results in an inverted expression of *Fgf3/10* in labial and lingual mesenchymes, resulting in an expanded LiCL and lingual ameloblast formation, with smaller LaCL and an abnormal development in labial ameloblasts [106]. Moreover, a hypomorphic *Bcl11b* mutation has shown to induce the proliferation of adult TA cells and to maintain the quantity of epithelial stem cells. Yet, whether this mechanism includes FGF3 remains unknown [131]. On the other hand, TBX1 induces the proliferation of incisor epithelial cells by inhibiting the transcriptional activity of PITX2, which in turn supports the expression pattern of p21, a cell cycling inhibitor [132]. Supporting this view, incisors of *Tbx1*-deficient mutants cultured in kidney capsules exhibit hypoplasia and complete lack of enamel [91].

The expression of E-cadherin is negatively regulated by FGFs in the stem cells, which causes these cells to migrate out of the niche, followed by proliferation and differentiation into TA cells, which can become ameloblasts afterwards. In *Fgf3^−/−^;Fgf10^+/−^* mice, no downregulation of E-cadherin expression is detected in the TA region, while cell proliferation decreases dramatically [127]. However, an abnormal expression of *Fgf3* has been found in the lingual side of the mesenchyme in *Spry2^+/−^;Spry4^−/−^* mice, which in turn leads to the formation of TA cells and ameloblasts without lingual E-cadherin [93, 127].

The *Shh* expression is partly regulated by *Fgf9* in the epithelium. The mice exhibit a reduction in the size of the labial cervical loop, where the *Shh* expression area expands to a more posterior location due to the deletion of *Fgf9* [72]. *Shh* mRNA expression is significantly downregulated by ectopic FGF9 in incisor explants [72]. Given the essential role of TA region *Shh* expression in ameloblast differentiation [133], FGF9 may take part in protecting progenitor cells from the *Shh* signal so as to keep them undifferentiated in the cervical loop. This would be parallel to the forming limb, where *Etv4/5* dependent on FGF is necessary to repress *Shh* expression in the mesenchyme of the anterior limb bud and limit *Shh* expression posteriorly [134, 135]. Yet, it is not clear whether Etv family molecules have similar roles during the development of the incisor.

BMP4 and activin, two proteins from the TGF*β* family, modulate the activity of FGF and the regulation of the asymmetry of the incisor during incisor development. The symmetrical expression of BMP4 occurs throughout the mesenchyme and suppresses the expression of *Fgf3* indirectly in the lingual mesenchyme. The expression of activin is more robust in the labial mesenchyme, and the bead implantation study in incisor explants at E16 indicates that activin offsets the effect of BMP4 [80]. This can maintain the expression of *Fgf3* on the labial side of the mesenchyme and in turn increase the proliferation of stem cells. In addition, the activity of residual activin on the lingual side is counteracted by follistatin that was detected in the lingual epithelium and functions to preserve the effect of BMP4 on repressing the *Fgf3* expression in the lingual mesenchyme. Consequently, embryos without the *Fst* gene which encodes follistatin have shown to exhibit ectopic expression of *Fgf3* in the lingual mesenchyme; these results in the expanded LiCL and lingual ameloblasts as well as enamel formation [80]. On the contrary, *Fst* misexpression in the epithelium leads to a reduction in the expression of *Fgf3* and subsequently causes reduced proliferation and the size of LaCL [80]. BMP4 can also increase the differentiation ability of ameloblasts in the more distal side of the labial epithelium, while in the lingual epithelium this process is repressed by follistatin expressed locally to maintain the asymmetry of the incisor [136]. Coincident with the view that BMP4 acts in two regions of the incisor during its development, misexpression of noggin (the inhibitor of BMP) leads to incisor hyperplasia because in the cervical loop the proliferation of the population of progenitor cells is promoted. However, as ameloblast differentiation normally promoted by BMP signaling is inhibited, the incisors do not form enamel in the mutant [137]. Furthermore, mesenchymal TGF*β* receptor type I (Alk5/Tgfbr1) can modulate the proper initiation of tooth and the epithelium development of the incisor [138, 139]. Mesenchymal *Fgf3* and *Fgf10* expressions were downregulated when *Alk5* was knocked out specifically in the mesenchyme, causing fewer label-retaining cells and decreased proliferation in the cervical loop. Exogenous FGF10 proteins could rescue this phenotype in incisor explant culture [138]. The mesenchymal expression of *Fgf* is partially activated via transcription factors MSX1 and PAX9, which can initiate *Fgf3* and *Fgf10* by E12.5 and in turn contribute to subsequent incisor development [128, 139, 140]. Moreover, with epithelial deletion of *Isl1*, FGF signaling is upregulated and is associated with both lingual cervical loop-generated ectopic enamel and labial side premature enamel formation [141]. FGF signaling and downstream signal transduction pathways are also suppressed in *Ring1a^−/−^;Ring1b^cko/cko^* incisors [142].

It has also been reported that FGF signaling is required for stem cell self-renewal and can prevent differentiation of dental epithelial stem cells (DESCs) in the cervical loop and in the DESC spheres. The inhibition of the FGF signaling pathway can decrease proliferation and increase apoptosis of the cells in the DESC spheres. On the other hand, inhibiting FGFR or its downstream targets can decrease *Lgr5*-expressing cells in the cervical loop and induce cell differentiation in both cervical loop and the DESC spheres [143]. In addition, FGF signaling may also be required for YAP-induced proliferation in T-A cells [144].

## 8. The Importance of FGF Signaling in Human Tooth Development

It has been shown that in clinics, FGFs are required for human tooth development. Its dysregulation seriously affects tooth development in humans, leading to enamel defects and tooth agenesis. Lacrimo-auriculo-dento-digital (LADD; Online Mendelian Inheritance in Man (OMIM) database no. 149730) syndrome, a congenital autosomal dominant disorder, results from the heterozygous missense mutations in *FGF10*, *FGFR2*, and *FGFR3*. LADD is characterized by aplasia, hypoplasia/atresia of salivary/lacrimal glands, ears with cup shape, and hearing loss [145–148], as well as various dental phenotypes, including hypodontia, teeth with peg shape, and hypoplastic enamel [149]. In addition, compound heterozygous or homozygous *FGF3* mutations cause congenital deafness with labyrinthine aplasia, microtia, and microdontia (LAMM; OMIM no. 610706) syndrome which is also characterized by malformed external ear, malformed/missing inner ear, and peg-shaped teeth with reduced size [150–152].

Mutations in *FGFRs* can also cause several syndromes such as Apert and Crouzon syndromes. Among them, the Apert syndrome (OMIM no. 101200) derives from gain of function in *FGFR2* mutations and is characterized by hypoplasia of midface, craniosynostosis, and syndactyly of the hands and feet [153]. The mutations in *FGFR2* can cause Crouzon syndrome (OMIM no. 123500) characterized by craniosynostosis, leading to hypertelorism, prognathism of mandible, hypoplastic maxillary, and short upper lip [154]. Patients with Apert and Crouzon syndromes usually exhibit hypodontia, mostly of the third molar, second incisor in maxillary, and second premolar in mandible [155, 156].

It has also been reported that the application of FGF2 can promote the regeneration of periodontal tissues [157, 158]. In this study, a clinical trial was performed in 253 adult periodontitis patients. A modified Widman periodontal surgery was carried out, and during the surgery, a 200 *μ*L investigational formulation containing FGF2 in different concentrations was applied to 2- or 3-walled vertical bone defects. The application of FGF2 showed a significant effect over the placebo-control group (*p* < 0.01) for the bone fill percentage after 36 weeks of administration. The results demonstrate that topical FGF2 application can treat the bone defect caused by periodontitis and it can be efficacious in human periodontal tissue regeneration [158]. In addition, FGF2 can also promote the neovascularization of human dental pulps which is severed [159]. Human molars without caries were used for preparation of tooth slices which were then treated with 0–50 ng/mL recombinant human FGF2 for a week in vitro. The result showed that the density of microvessel in dental pulps was enhanced with FGF2 treatment compared with untreated controls, indicating that topical application of FGF2 in advance of replantation might be efficacious in the treatment for avulsed teeth [159]. Another study isolated and characterized stem cells from inflamed pulp tissue of human functional deciduous teeth (iSHFD) in order to investigate the role of FGF2 on the potential of regeneration of these cells [160]. Application of FGF2 to iSHFD during their expansion improved the colony-forming efficiency of the cells and increased their potential of migration and proliferation, but decreased their potential of differentiation in vitro. This provides a good stem cell source for future applications in clinics and a new way to use inflamed tissues which has to be discarded before.

Given the results of these studies, the application of FGFs can be a potential treatment for human dental diseases, even for those defects in tooth development as well as for the syndromes caused by mutations in *FGFs* and *FGFRs*. The delivery of FGFs to the primary nidus still needs to be improved, and further clinical trials are also required.

## 9. Conclusion

FGF signaling has been the focus of intense interest over the past years, and thus, it has been investigated both in vitro and in vivo, by using different cell and genetic mouse models. The FGF expression has an important role in different stages of tooth development, including tooth initiation and mineralized tissue formation. Uniquely in rodents, FGFs are essential to maintaining the stem cell niche fueling the unceasingly growing incisor throughout their lifetime. The tooth offers an attractive model to further dissect the regulation and transduction of FGFs in developmental as well as stem cell biology. Despite the understanding of the role of FGF signaling, many questions remain unexplored. Thus, it is necessary to further investigate more molecular mechanisms which regulate FGFs and examine their other pathways. In addition, like the irreplaceable function of FGFs in regeneration and tissue homeostasis in the mouse model, FGFs have also been found to be involved in these processes in humans. By controlling the activity of FGFs, it could be possible to obtain novel methods to treat human diseases. Studies on the underlying mechanism of FGF regulation in teeth may potentially extend the current knowledge of other organ systems and may also offer insights into progression of diseases, presenting new therapeutic approaches.
